# Persistence of single species of symbionts across multiple closely-related host species

**DOI:** 10.1038/s41598-019-54015-2

**Published:** 2019-11-25

**Authors:** Jorge Doña, Carolina Osuna-Mascaró, Kevin P. Johnson, David Serrano, Raül Aymí, Roger Jovani

**Affiliations:** 1 Department of Evolutionary Ecology, Estación Biológica de Doñana (EBD-CSIC), Avda. Americo Vespucio 26, Sevilla, 41092 Spain; 2 AllGenetics & Biology SL, Edificio CICA, Campus de Elviña s/n, 15008 A Coruña, Spain; 3 Department of Genetics, Faculty of Science, University of Granada, Avda. Fuentenueva s/n, Granada, 18071 Spain; 4 Illinois Natural History Survey, Prairie Research Institute, University of Illinois at Urbana-Champaign, 1816 S. Oak St., Champaign, IL 61820 USA; 5 Department of Conservation Biology, Estación Biológica de Doñana (EBD-CSIC), Avda. Americo Vespucio 26, Sevilla, 41092 Spain; 6 Institut Català d’Ornitologia, Museu de Ciències Naturals de Barcelona, Pl. Leonardo da Vinci, 4-5, a, Barcelona, 08019 Spain; 7 Present Address: Illinois Natural History Survey, Prairie Research Institute, University of Illinois at Urbana-Champaign, 1816 S. Oak St., Champaign, IL 61820 USA

**Keywords:** Biodiversity, Coevolution

## Abstract

Some symbiont species are highly host-specific, inhabiting only one or a very few host species, and typically have limited dispersal abilities. When they do occur on multiple host species, populations of such symbionts are expected to become genetically structured across these different host species, and this may eventually lead to new symbiont species over evolutionary timescales. However, a low number of dispersal events of symbionts between host species across time might be enough to prevent population structure and species divergence. Overall, processes of evolutionary divergence and the species status of most putative multi-host symbiont systems are yet to be investigated. Here, we used DNA metabarcoding data of 6,023 feather mites (a total of 2,225 OTU representative sequences) from 147 infracommunities (i.e., the assemblage consisting of all mites of different species collected from the same bird host individual) to investigate patterns of population genetic structure and species status of three different putative multi-host feather mite species *Proctophyllodes macedo* Vitzthum, 1922, *Proctophyllodes motacillae* Gaud, 1953, and *Trouessartia jedliczkai* (Zimmerman, 1894), each of which inhabits a variable number of different closely related wagtail host species (genus *Motacilla*). We show that mite populations from different host species represent a single species. This pattern was found in all the mite species, suggesting that each of these species is a multi-host species in which dispersal of mites among host species prevents species divergence. Also, we found evidence of limited evolutionary divergence manifested by a low but significant level of population genetic structure among symbiont populations inhabiting different host species. Our study agrees with previous studies showing a higher than expected colonization opportunities in host-specific symbionts. Indeed, our results support that these dispersal events would allow the persistence of multi-host species even in symbionts with limited dispersal capabilities, though additional factors such as the geographical structure of some bird populations may also play a role.

## Introduction

Symbiont organisms tend to be highly adapted to live on their native hosts^1–4^. One might expect that the level of specialisation of symbionts would drive symbiont species to be highly host-specific (i.e., to inhabit a single host species, in the most extreme scenario)^5–10^. However, symbiont species inhabiting more than a single host species are relatively frequent across different groups of symbionts^1,2^.

In general, populations of symbionts inhabiting different host species are expected to become genetically structured (or sorted) by host species and to lead to new symbiont races and species over evolutionary timescales^8,11–16^. Among the agents contributing to the divergence process, the symbiont mode of transmission (i.e., vertical vs horizontal) is one of the main factors contributing to symbionts population structure and divergence^2,15,17,18^. Vertical transmission, in which symbionts are transmitted from parents to offspring, is only possible between symbiont populations inhabiting a particular host species (though it may be possible if a hybridisation event occurs^19^). Thus, populations of symbionts whose main mode of transmission is vertical are expected to become quickly structured by host species and eventually speciate on that host species^1,2,17,20–23^. Alternatively, in horizontally transmitted symbionts, dispersal goes from one host species to another and tends to erode the population genetic structure among-host populations^1,2,17,20–23^. Thus, populations of symbionts with an elevated rate of horizontal transmission are expected to exhibit lower among-host population structure, not necessarily speciating on each host species^1,2,17,20–23^.

Highly host-specific symbionts, in which vertical transmission is generally the main mode of dispersal, are however known to contain multi-host species (e.g., Doña *et al*.^24^). One explanation for this pattern is that despite vertical transmission being the main mode of dispersal, dispersal events (either periodic or episodic) among populations on different host species might be enough to prevent populations from becoming genetically structured and diverging. Thus, these dispersal events would sustain multi-host species in host-specific symbionts^2^. Indeed, some recent studies have indicated that colonisation opportunities may be underestimated for some of these symbionts with limited transmission capabilities, and clade-limited host-switching may occur frequently^25–28^. Alternatively, multi-host symbiont species from relatively host-specific symbionts could represent undiscovered cryptic species^29–33^. Indeed, cryptic species might be common in small-bodied symbionts inhabiting closely related hosts, because these hosts can offer a similar habitat, and therefore, there might be strong selective pressure for highly similar symbiont phenotypes^34^. Overall, the population structure and species status of multi-host species of most host-specific symbiont systems are yet to be well understood.

Feather mites are host-specific symbionts with limited dispersal capabilities, but also with some clade-limited host-switching^25–27,35–39^. Previous studies have documented multi-host species^35–37,40^ and cases of morphologically-cryptic but genetically different mite species^35–37,40^. Also, genetic structure has been observed (1) among populations of mites at the level of infrapopulations^41^, (2) among feather mite populations inhabiting different bird host species^35–37,40^ and (3) among different populations of the same passerine bird species^41^. Most putative multi-host bird-mite systems remain, however, unexplored. Large scale studies, which might allow precise assessments of population genetic structure and species status^41^, are nonexistent.

Here, we investigated the process of evolutionary divergence and species status of populations of feather mite multi-host species in the genera *Proctophyllodes* and *Trouessartia*; each of which inhabits different closely related species of European wagtails hosts (genus *Motacilla*). Specifically, we used DNA metabarcoding data from 6,023 individual mites (a total of 2,225 OTU representative sequences) representing 146 infracommunities of *Proctophyllodes motacillae*, *Trouessartia jedliczkai*, and *Proctophyllodes macedo* inhabiting the white wagtail *Motacilla alba* Linnaeus, 1758, western yellow wagtail *Motacilla flava* Linnaeus 1758, and grey wagtail *Motacilla cinerea* Tunstall, 1771.

## Results

Final mitochondrial COI sequence alignments contained 516 sequences of 388 bp for *P. macedo*, 680 sequences of 390 bp for *P. motacillae*, and 1,029 sequences of 388 bp for *T. jedliczkai*.

**Figure 1:**
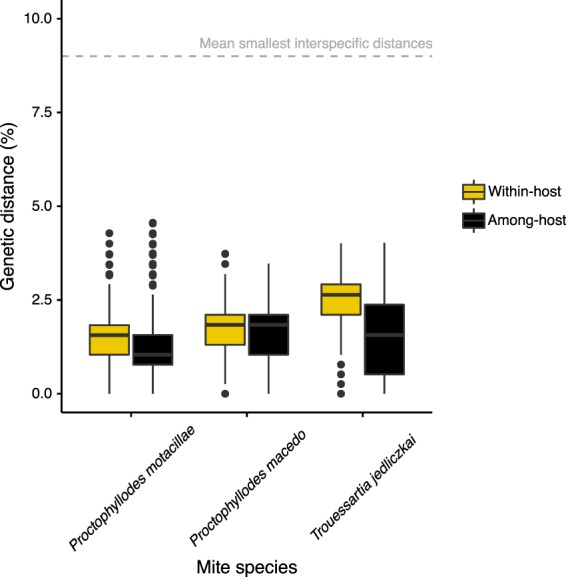
Boxplots showing within and among host infrapopulation genetic distances of the different mite species. Dashed grey line shows a reference interspecific threshold for feather mites^35^.

For *P. motacillae*, *P. macedo*, and *T. jedliczkai*, species delimitation analyses each supported the existence of a single mite species despite inhabiting different number of host species. First, when evaluating whether mite populations on different host species could be different species, we consistently found an overlap between within host - and among host infrapopulations genetic distances (Fig. 1). Accordingly, we did not find any suitable threshold for “species” delimitation nor high percentages of successful identifications when using the best close match method (38%, 32%, and 32% for *P. motacillae*, *P. macedo*, and *T. jedliczkai*, respectively; Fig. S1). Second, for each mite species studied, we found a single partition (i.e., a single hypothetical species) in the ABGD analyses.

**Figure 2:**
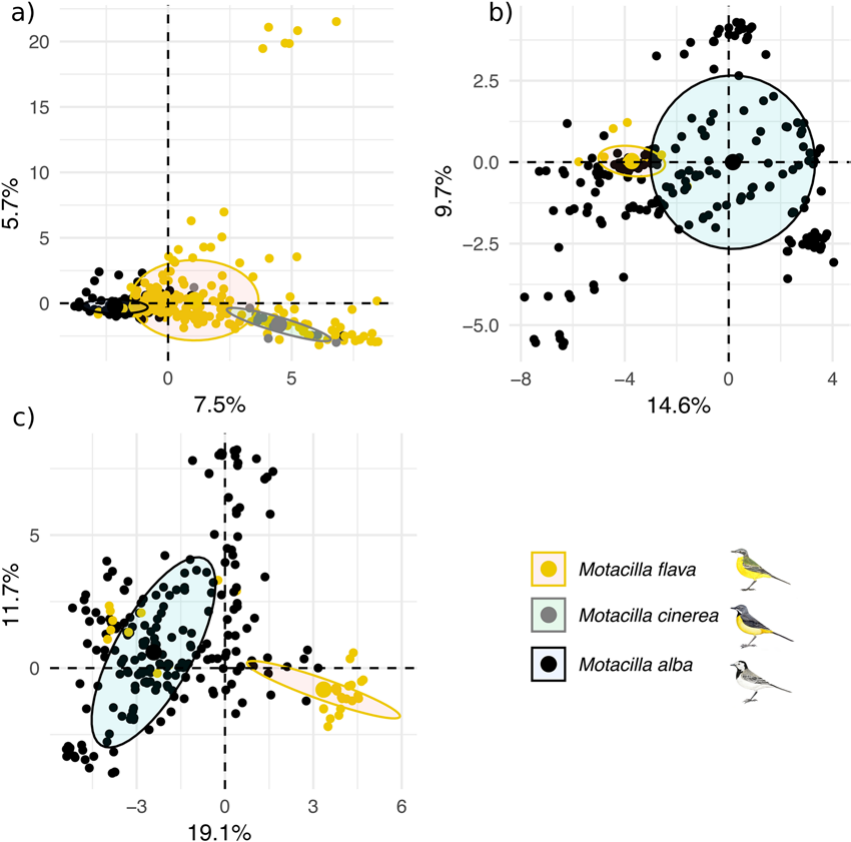
Principal Component Analysis results (PCA) of the genetic structure among mites species: (**a**) *P. motacillae*, (**b**) *P. macedo*, and (**c**) *T. jedliczkai*. Each point represents one representative mite sequence, and points are coloured by host species.

Population genetic analyses revealed variable but overall low levels of genetic structure among symbiont populations inhabiting different host species. AMOVA and PCA analyses showed that most of the variation was due to the intragroup variation (70%, 73%, and 52% for *P. motacillae*, *P. macedo*, and *T. jedliczkai*, respectively; *P* < 0.01 in all cases; Fig. 2, Table 1). Also, most of the major haplotypes were confined to a single host species (Fig. S2). In all mite species, we found a low, but still substantial, number of haplotypes shared between mite infrapopulations on different host species. Specifically, we found ten *P. motacillae* haplotypes shared between *M. alba* and *M. flava*, five *T. jedliczkai* haplotypes shared between *M. alba* and *M. flava*, three *P. macedo* haplotypes shared between *M. alba* and *M. flava*, and one *P. motacillae* haplotype shared between *M. flava* and *M. cinerea*. Lastly, in all mite species except *T. jedliczkai*, we did not find haplotypes significantly clustered by host species in haplotype networks (Fig. S2).

**Table 1:** AMOVAs of mites populations at between and within host species levels using representative sequences (i.e., one sequence per OTU, and more than one OTU could be called into each infrapopulation) of mite infrapopulations. Statistically significant values are asterisked.

	P. macedo				P. motacillae				T. jedliczkai			
	d.f.	Var. comp.	% Var.	P	d.f.	Var. comp.	% Var.	P	d.f.	Var. comp.	% Var.	P
Between hosts	1	1.93	26.67	<0.001*	2	1.62	30.40	<0.001*	1	4.16	48.53	<0.001*
Within hosts	515	5.32	73.32		678	3.71	69.59		1028	4.41	51.46	
Total	516	7.25	100.00		680	5.33	100.00		1029	8.57	100.00	

Nucleotide and haplotype diversity values were high for most species and populations but differed between mite populations of the same mite species inhabiting different hosts (Table 2). These differences, however, did not follow an apparent host-driven pattern across mite species (Table 2, Fig. S2). Also, in most mite populations on different host species, we found signatures of population expansion evidenced by small, negative Tajima’s D values, small, positive R^2^ values, and star-shaped haplotype networks (Table 2, Fig. S2).

**Table 2:** Number of infrapopulations (n), nucleotide diversity (π), haplotype diversity (Hd), Tajima’s D (D), Tajima’s D P value, R^2^, and R^2^ P value of mite populations.

	M. alba							M. flava							M. cinerea						
	n	π	Hd	D	P	R^2^	P	n	π	Hd	D	P	R^2^	P	n	π	Hd	D	P	R^2^	P
P. motacillae	15	0.008	0.995	−2.476*	0.013	0.014*	0.002	75	0.013	0.995	−1.970*	0.048	0.024*	0.044	5	0.009	0.995	−0.887	0.37	0.090*	0.09
P. macedo	30	0.016	0.995	−0.914*	0.041	0.022*	0.037	7	0.008	0.996	−0.914	0.36	0.090	0.10							
T. jedliczkai	38	0.017	0.993	−2.086*	0.036	0.020*	0.027	8	0.009	0.993	−2.774*	0.005	0.004*	0.004							

## Discussion

For each of the three multi-host feather mite species distributed across three closely related host species, species delimitation analyses supported the existence of a single species inhabiting different host species. Our results agree with previous morphological assessments carried out on these mite species inhabiting these bird species (Doña *et al*.^24^ and references therein). Notably, however, in all the symbiont species studied, we found evidence of population divergence manifested by a low but significant level of population genetic structure among symbionts populations inhabiting different host species. Altogether, these results add to previous findings suggesting that feather mites can potentially disperse among host species more than expected based on their known biology, and suggest the presence of small scale gene flow between populations of feather mites on different host species^24,25,27^. Nevertheless, additional factors such as the geographical structure of some bird populations, introgression, or incomplete lineage sorting may be playing a role in explaining some of the patterns found here (e.g., *P. macedo* and *T. jedliczkai* haplotype distributions; Fig. S2)^16,42^.

The levels of genetic structuring found for these multi-host mite species are higher than those found in multi-host symbionts with higher dispersal capabilities, e.g., this study: F_st_ (Phi_st_) = 0.26–0.48 vs 0.01–0.20 between populations of conspecific lice on different host species^17,18^. This result supports that differences in dispersal ability shape patterns of population structure and evolution^17–19^. In the same vein, the degree in which mite populations were structured by host species was variable across the three mite species, thus again supporting that symbiont-specific traits such as dispersal capabilities (e.g., phoretic vs non-phoretic or strict vertical vs horizontal dispersal) might influence genetic structuring patterns^18,23,37,43–45^. Specifically, some of the patterns of genetic structuring found here (see below), even though not directly comparable, are similar to those of previous studies on feather mite species from the same genera^32^. In particular, we found that *Trouessartia* populations were more structured than those of *Proctophyllodes*, as was previously found in two different species of the same genera, e.g., 50% vs 19% of the variation explained by the among-infrapopulation genetic structure component in *Trouessartia bifurcata* (Trouessart) vs *Proctophyllodes sylviae*^32^. Nonetheless, contrasting effective population sizes^46^ or processes such as hybridisation^19^ may distort genetic estimates from a single-locus such as the COI used in this study, and therefore, studies using multilocus population genomic approaches (e.g., SNPs^15^) are encouraged.

Different non-mutually exclusive scenarios can explain the dispersal events that have occurred or still do occur between host species, and that are behind the existence of single species of mites across these host species. First, mites may have recently colonised these host species, and hence they may not have diverged yet (i.e., a recent host switch without ongoing gene flow). These colonisations may have occurred at different times, and therefore, explain the differences in the degree of genetic structuring among mite species. However, they could have also colonised these different host species nearly simultaneously, and thus the differences in genetic structuring may be the consequence of mite-specific traits (e.g., a species-specific dispersal rate). Small populations are especially vulnerable to genetic drift^47^, and this applies particularly to mitochondrial markers because of their lower effective population size compared to diploid nuclear loci, as well as to symbionts colonising a new host species, because only a few haplotypes are likely found in new populations^21,48–50^. Therefore, under a recent colonisation scenario, we might expect low levels of genetic diversity, and multiple recent haplotypes to be descendant from a clustered pool of ancestral haplotypes^32,47^. Instead, we found that genetic diversity was relatively high for most within-host populations, and multiple haplotypes were shared all along the haplotype networks. Furthermore, mite species did not consistently exhibit higher or lower genetic diversity values on particular host species (Table 2), and some within-host populations (e.g., *T. jedlizckai* in *M. flava*) showed low levels of genetic diversity and a star-shaped haplotype network with multiple recent haplotypes descended from a single common ancestral haplotype (Table 2, Fig. S2).

Second, mites may have been associated only with one of these or another wagtail species^24^ and colonised different *Motacilla* species afterwards while maintaining gene flow with the source population (i.e., incomplete host-switching^51^). If an incomplete host switch has occurred, we would expect an increase in the population size of the colonising symbiont^32^. Interestingly, we have found evidence of population growth in all the populations studied (Table 1, Fig. S2). These population growth signatures may support a scenario of incomplete host-switching^32^, which may also be following recent studies on the dynamism of bird feather mites associations^25,27,37,52^.

Finally, it is possible that these mites were inherited from the common ancestor of all these wagtail species and maintained gene flow among diverging host species as the wagtail species were diverging from each other. Indeed, this scenario could be supported by the fact that all these wagtail hosts diverged very recently from each other^53^. Furthermore, even low levels of gene flow^54–56^ among populations of mites on different host species could be enough to prevent species divergence (i.e., failure to speciate^20^). In a failure to speciate scenario, the symbiont population size is expected to remain constant. Hence, the signatures of population growth that were observed in all species of mites do not support this scenario^32^. However, over long time periods, severe bottlenecks are expected given the population dynamics of these symbionts, and population growth after bottlenecks may also explain the signatures found in the demographic tests^37,52^.

## Materials and Methods

We followed the sampling protocol and the DNA metabarcoding pipeline of Vizcaíno *et al*.^57^ and Doña *et al*.^27^. In brief, we collected feather mites from live individuals of *M. alba*, *M. flava*, and *M. cinerea* in different localities in Spain (Table S1). A total of 6,023 mites from 146 feather mite infracommunities were collected from the host species *M. alba* (n = 2,660), *M. flava* (n = 3,255), and *M. cinerea* (n = 108) (Table S1). Mites were sampled with a flattened preparation needle or with an ethanol-impregnated cotton swab. Afterward, we extracted DNA from all the collected mites of each individual bird (i.e., infracommunity) using the HotSHOT method^58^. The ethanol was first evaporated, then a solution of NaOH 1-M was added to the dry wells, incubated at 95 °C and finally neutralized with equivalent amounts of Tris-Cl. For each infracommunity, we constructed the DNA amplicon libraries by amplifying a region of the mitochondrial COI gene in a two-step PCR (see Vizcaino *et al*.^57^ for details). We followed the Illumina protocol for bacterial 16S DNA metabarcoding. The 146 libraries were pooled together (i.e., multiplexed) and sequenced in one MiSeq PE300 run (MiSeq Reagent Kit v3).

After sequence pre-processing, we ended up with 1,394,138 sequences (statistics per sample, i.e., infracommunity: mean = 9,548; min = 109; max = 174,412) which were assigned to five different mite species (Table S1). *Proctophyllodes motacillae* was the most prevalent species [Number of infrapopulations (N) = 95; 65%], followed by *T. jedliczkai* (N = 46; 31%), and *P. macedo* (N = 37; 25%) (Table 1). *Pteronyssoides motacillae* Mironov, 1985 (N = 1; 0.7%) and *Proctophyllodes sylviae* Gaud, 1957 (N = 2; 1.4%) were also found but not included in further analyses because of their extremely low prevalence (Table S1).

The fastq reads were quality-checked with FastQC^59^. Then, we imported the reads into Geneious v.8.1.7^60^ for quality-trimming analyses. We trimmed a region of 36 and 120 bp from R1 and R2 reads, respectively, according to the average Phred score (minimum quality score of 28). The R1 and R2 files were then exported in FASTA format and were concatenated using the fuse.sh script available from the BBmap package v.37.00^61^. We only saved the concatenated sequences with the maximum possible length. To label the sequences with the sample identifier and merge them into a single file, we used the split_libraries.py script included in the pipeline of QIIME v.1.9.0^62^. Then, we selected the Operational Taxonomic Units (OTUs) with a 100% similarity threshold using a de novo clustering method and the UCLUST algorithm^63^. We also used a filter to eliminate or minimize mistagging events (e.g., only OTUs with more than 100 identical reads were kept), and after this, we selected the most abundant sequence of each OTU as the representative sequence of that OTU. We made the taxonomic assignment of each representative sequence using the assign_taxonomy.py script of QIIME. The assignment was done with the RDP classifier^64^ and a minimum confidence score of 96.6% against a reference database (Doña *et al*.^35^). Lastly, we aligned the representative sequences of each mite species using MAFFT v7.388^65^ with default parameters and refined the alignments with trimAL v1.2^66^ using the “gappyout” method.

Unless otherwise specified, all the analyses were done in R v3.5^67^. We conducted species delimitation analyses for the mite species *P. macedo*, *P. motacillae*, and *T. jedlizcai*. First, we used the local minima function of the package SPIDER v.1.3^68^ to estimate an optimal threshold for delimiting species. We also used the web version of ABGD^69^ to review our species hypotheses. We use the default settings on a set of prior minimum genetic distances ranging from 0.001 to 0.1. Lastly, we evaluated the accuracy in cataloguing mite sequences to putatively different host-specific species using the genetic distance-based method BCM^70^. We used the *bestCloseMatch* function of SPIDER and the genetic threshold previously calculated (see above).

We estimated the population genetic structure of these above-mentioned mite species among mite populations on different host species. First, we conducted analyses of molecular variance (AMOVA) using the *poppr.amova* function from POPPR v.2.8^71,72^, and we tested for statistical significance using the *randtest* function of ADE4 v.2.1^73–76^. We further investigated the genetic structuring among samples, based on genetic distances, through principal components analyses (PCA) with the *dudi.pca* function of ADE4.

Additionally, we estimated population genetic parameters using PEGAS v.0.1^77^. We used the *nuc.div* function to calculate nucleotide diversity (π, hereafter genetic diversity^78^). Also, we calculated parameters to detect population expansions. We calculated Tajima’s D^79^, using the *tajima.test* function; and estimated statistical significance assuming that D follows a normal distribution with mean zero and variance one. We also calculated R^2^ using the *R2.test* function and evaluated its statistical significance through 1,000 coalescent simulations. Finally, we estimated the haplotype diversity (Hd) using the *hap.div* function, and used the *haplotype* and *haplonet* functions to build haplotype networks and study the mites haplotype distribution.

## Supplementary information

Supplementary figures

Table S1

## Data Availability

The raw data and the representative sequences have been deposited in Figshare (10.6084/m9.figshare.7857647; https://figshare.com/s/38abf165a4e5c87ec26f).
