# Supplementary material for: Persistence of single species of symbionts across multiple closely-related host species
Source: Sci Rep. 2019 Nov 25;9:17442. doi: 10.1038/s41598-019-54015-2 (PMC6877549; doi:10.1038/s41598-019-54015-2)

Supplementary material for: *Persistence of single species of symbionts across multiple closely-related host species*

Jorge Doña, Carolina Osuna-Mascaro, Kevin P. Johnson, David Serrano Raül Aymí, and Roger Jovani

Figure S1. Density plots showing the results of the localMinima funct ion from the R package Spider (Brown et al., 2012).

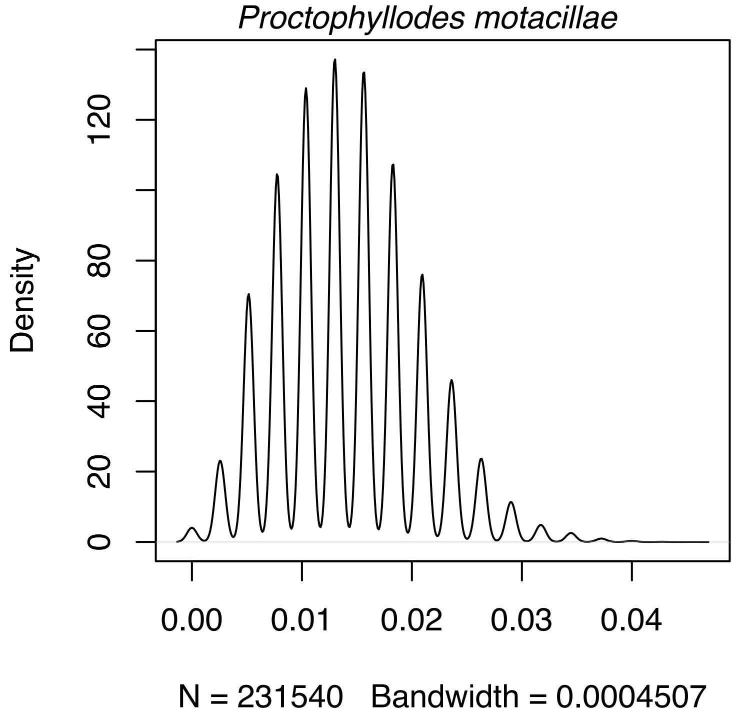

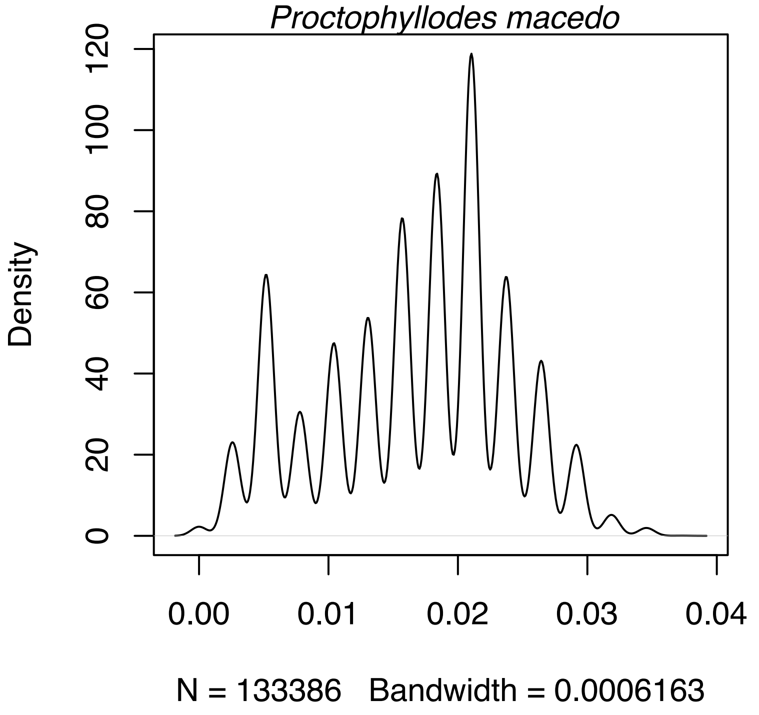

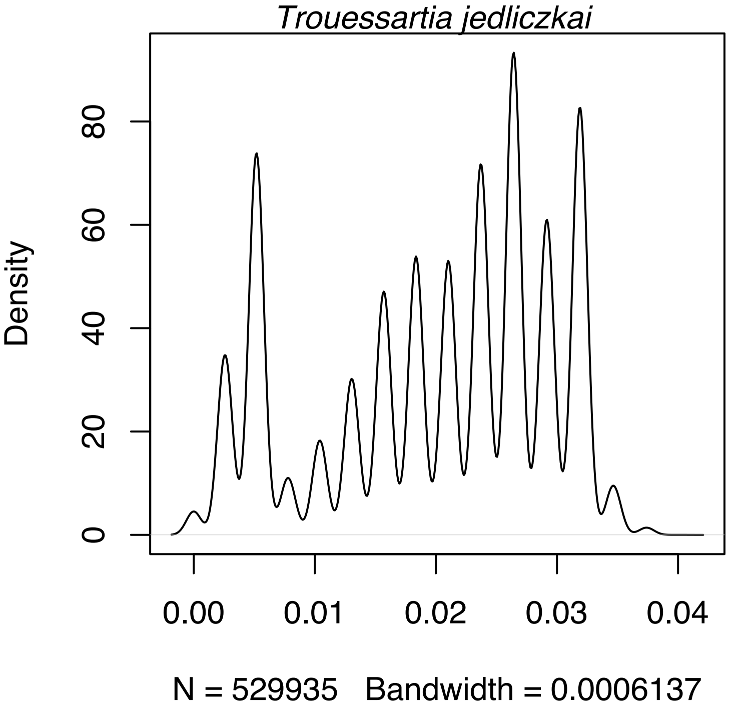

Figure S2. Haplotype networks of mite species. From left to the right: *P. motacillae*, *P. macedo* and *T. jedliczkai*. The size of the circles is proportional to the number of haplotype copies present in the alignment (an empty circle at the bottom of each network represents the size of one sequence). Colors depict hosts species. Black dots represent mutational steps.

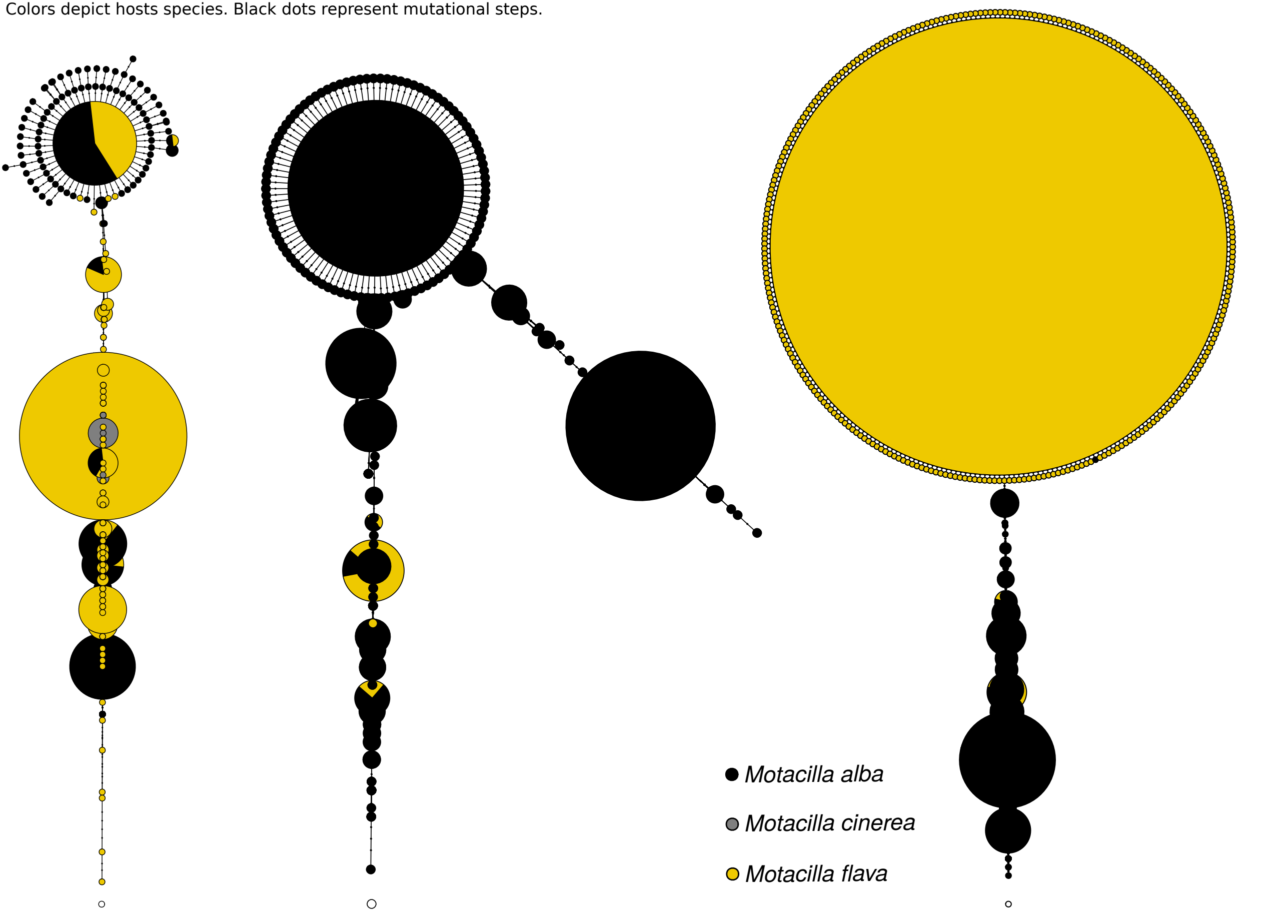

Supplement: Supplementary file 1 — Supplementary figures [file 41598_2019_54015_MOESM1_ESM.pdf]
